# Role of Iron Metabolic Disturbances and Inflammatory Iron Biomarkers in Liver Transplant Prognosis

**DOI:** 10.7150/ijms.113479

**Published:** 2025-07-01

**Authors:** Lu Yu, Jun Xu, Ting Que, Zhezhi Miao, Yifeng Zhou, Shuping Que, Shusen Zheng, Zhengtao Liu

**Affiliations:** 1 Key Laboratory of Artificial Organs and Computational Medicine in Zhejiang Province, Shulan International Medical College, Zhejiang Shuren University, Hangzhou, China.; 2 Shulan (Hangzhou) Hospital, Shulan International Medical College, Zhejiang Shuren University, Hangzhou, China.; 3 School of Medicine, Zhejiang Chinese Medical University, Hangzhou, China.; 4 Division of Hepatobiliary and Pancreatic Surgery, Department of Surgery, First Affiliated Hospital, School of Medicine, Zhejiang University, Hangzhou, China.; 5 NHC Key Laboratory of Combined Multi-organ Transplantation, Key Laboratory of the Diagnosis and Treatment of Organ Transplantation, School of Medicine, Chinese Academy of Medical Sciences, First Affiliated Hospital, School of Medicine, Zhejiang University, Hangzhou, China.; 6 Key Laboratory of Organ Transplantation, Zhejiang Province, First Affiliated Hospital, School of Medicine, Zhejiang University, Hangzhou, China.; 7 Guangxi Clinical Research Center for Birth Defects, Guangxi Key Laboratory of Birth Defects Research and Prevention, Guangxi Key Laboratory of Reproductive Health and Birth Defects Prevention, Maternal and Child Health Hospital of Guangxi Zhuang Autonomous Region, Nanning, China; 8 Ya-er-zhuang Clinics, Hangzhou, China.

**Keywords:** Iron Metabolism, Liver Transplantation, Ischemia-reperfusion injury, Biomarkers, Oxidative stress, Clinical management

## Abstract

Iron metabolism plays a pivotal role in liver transplantation, significantly impacting outcomes for both donors and recipients. The liver, central to iron homeostasis, is often impaired in chronic liver diseases leading to metabolic disorders that exacerbate liver damage. Liver transplantation (LT) is a critical treatment for end-stage liver diseases, with iron status in both donors and recipients influencing post-transplant outcomes. Studies indicate that pre-transplant iron overload in recipients is associated with poor liver function recovery, increased graft rejection risk, and reduced patient survival. The iron metabolic state of donors also affects the functionality of the transplanted liver, impacting transplant success and patient prognosis. Biomarkers such as hepcidin, serum ferritin, and total iron-binding capacity are significant predictors of LT prognosis, yet their specific roles and impacts remain inconclusive. This review systematically assesses how variations in iron metabolic levels of donors and recipients affect patient outcomes following LT, aiming to optimize iron metabolism regulation in clinical management to enhance transplant success, reduce postoperative complications, and improve long-term patient quality of life. Future research should focus on developing personalized iron metabolism management protocols to refine these approaches and enhance transplant care.

## Introduction

Iron, an essential trace element for human physiology, is distributed in a tightly regulated manner across the body. Approximately 70% of total body iron is incorporated into hemoglobin within erythrocytes, 25% is stored as ferritin and hemosiderin in the reticuloendothelial system (primarily liver and spleen), with the remaining 5% existing in myoglobin, iron-containing enzymes, and plasma transferrin-bound forms^1^. The liver, as the central hub of systemic iron homeostasis, not only stores 1-1.5 g of iron but also orchestrates iron circulation through hepcidin secretion^2^.

Iron absorption occurs predominantly in the duodenum, where enterocytes utilize divalent metal transporter 1 (DMT1) for apical uptake and ferroportin for basolateral export, a process regulated by hepatic hepcidin through its interaction with ferroportin^3^. Under physiological conditions, daily iron absorption (1-2 mg) precisely compensates for basal losses through epithelial cell desquamation (0.6 mg/day), biliary excretion (0.3 mg/day), and minimal urinary elimination^4^. This delicate balance is disrupted in chronic liver diseases, where impaired hepcidin synthesis leads to paradoxical iron overload despite normal intake^5^.

The liver is the central organ for iron metabolism in the body, responsible not only for iron storage but also for regulating the metabolic balance of iron throughout the body by secreting hepcidin. Dysregulations in iron metabolism plays a crucial role in the progression of chronic liver diseases by modulating oxidative stress, inflammatory responses, and apoptosis^6^, ^7^. Hepcidin modulates the absorption and release of iron, maintaining a dynamic balance of iron within the body^8^.

However, liver function impairment, especially chronic liver diseases such as cirrhosis or fibrosis, often leads to iron metabolism disorders. This disorder includes both iron overload and iron deficiency, which can further exacerbate liver damage, forming a vicious cycle^9^. Previous studies have shown that iron overload is closely associated with the progression of certain liver diseases, further highlighting the complex relationship between liver function and iron metabolism^10^. Our systematic review and meta-analysis showed a dose-response relationship between serum ferritin and dietary iron intake with the incidence of metabolic syndrome and metabolic-associated fatty liver disease(MAFLD), highlighting the importance of iron metabolism in the pathogenesis of these conditions^11^.

Chronic liver disease is a significant threat to human health with severe complications and reduced life quality^12^, ^13^. Liver transplantation (LT) is often a life-saving therapeutic approach utilized with increasing frequency in the treatment of end-stage liver diseases^14^. The iron metabolic status of both the donor and recipient can influence outcomes before and after transplantation. Studies have demonstrated that pre-transplant iron metabolic disorders in recipients, such as iron overload, may lead to poor recovery of liver function post-transplant, an increased risk of graft rejection, and an impact on long-term patient survival rates^15^. Concurrently, the iron metabolic state of the donor may also affect the functional performance of the transplanted liver, thereby influencing the success rate of the transplant and the prognosis for the patient.

Biomarkers associated with iron metabolism, including hepcidin, serum ferritin, and total iron-binding capacity, have become significant predictors of liver transplant prognosis and the occurrence of complications^16^. However, research on the specific roles and impacts of these markers in donors and recipients remains limited, and the findings are not yet conclusive. Therefore, further investigation into the effects of iron metabolic levels in donors and recipients on the prognosis of liver transplantation holds substantial clinical relevance.

Several clinical studies have revealed that abnormal iron status in both donors and recipients—particularly elevated serum ferritin or low transferrin saturation—is independently associated with poor short- and long-term liver transplant outcomes, including increased risk of infection, delayed graft function, prolonged ICU stay, and reduced survival^15^, ^17^, ^18^.

The purpose of this review is to systematically examine existing clinical studies to assess how variations in iron metabolic levels of donors and recipients affect patient outcomes following liver transplantation. Understanding this process can aid in optimizing the regulation of iron metabolism in the clinical management of liver transplants, thereby enhancing the success rate of transplants, reducing postoperative complications, and improving the long-term quality of life for patients.

## Iron Metabolism is Closely Related to the Process of Liver Transplantation

Growing clinical evidence supports the involvement of iron metabolism in determining liver transplant prognosis. In a large retrospective study (n=328), Weismüller et al. found that high pre-transplant ferritin and low transferrin saturation in recipients were independently associated with reduced post-transplant survival^18^. Another prospective cohort study by Chow et al. (n=128) reported that elevated ferritin and hepcidin were predictive of post-transplant infections and adverse events^15^. Additionally, a Japanese study demonstrated that high ferritin levels in donor livers correlated with increased ischemia-reperfusion injury and early graft dysfunction^19^. These findings underscore the prognostic significance of donor and recipient iron status in clinical transplant settings (see Table 1).

### Effect of donor iron metabolism level on the prognosis of transplantation

#### Serum ferritin

Serum ferritin (SF), a pivotal iron-storage protein, is widely distributed across various human tissues, with particularly high concentrations in hepatocytes and reticuloendothelial cells. It plays a significant role in iron metabolism, liver diseases, and the prognosis following liver transplantation^20^.

Iron overload, as indicated by elevated serum ferritin, can initiate a cascade of events leading to oxidative stress. The excess iron acts as a catalyst in the production of reactive oxygen species (ROS). These ROS have the potential to cause severe damage to liver cells. In the context of the transplanted liver, this oxidative damage can manifest as early-onset graft dysfunction^17^. For instance, a study on liver transplant recipients demonstrated that those who received livers with serum ferritin levels exceeding two times the upper limit of normal had a notably higher incidence of cholestasis within the first week after transplantation. The high iron levels triggered the peroxidation of lipid membranes in hepatocytes, disrupting the normal bile flow and subsequently resulting in jaundice and elevated liver enzyme levels. In another case series, patients with elevated donor liver ferritin levels exhibited a 30% increased risk of developing hepatocyte necrosis in the early postoperative period compared to those with normal levels.

High serum ferritin levels have been implicated in an increased risk of infections following liver transplantation^21^. Iron is a vital nutrient for many microorganisms, and excessive iron availability creates a conducive environment for the growth of bacteria and fungi. This can potentially lead to septic complications and a poorer overall prognosis^22^. A retrospective analysis of transplant patients revealed that those with high preoperative donor liver ferritin levels had a 40% higher rate of bacterial infections, especially those caused by Gram-negative bacteria, within the first month after transplantation. The excess iron in the liver and bloodstream was found to enhance the virulence and growth of the infecting organisms, making it more challenging to control the infections^23^. This, in turn, led to longer hospital stays and, in some cases, increased mortality rates.

Another study noted that the preoperative iron metabolic status of liver transplant recipients substantially affects the postoperative ICU length of stay. Compared to the non-iron deficiency group, the iron deficiency group (serum ferritin < 100μg/L or 100 - 300μg/L and transferrin saturation < 0.20) had a significantly higher intraoperative transfusion of red blood cells and fresh frozen plasma and a significantly longer ICU length of stay^18^. Moreover, serum ferritin levels were significantly negatively correlated with ICU length of stay^24^.

In liver transplant donors, high serum ferritin levels may suggest hepatic iron accumulation, which increases the risk of oxidative stress post-transplantation. Excessive iron can cause membrane, mitochondrial, and DNA damage to the transplanted liver by promoting the generation of ROS, thereby delaying liver function recovery and augmenting the risk of postoperative complications^19^. Furthermore, elevated serum ferritin levels may exacerbate immune and inflammatory responses in the transplanted liver, heightening the risk of graft rejection^25^.

Clinical studies have established that patients with lower serum ferritin levels in transplant donors generally experience a more favorable prognosis, with smoother recovery of liver function post-transplantation. In contrast, donors with excessive serum ferritin levels may encounter a worse prognosis, including chronic graft dysfunction (CGD) and reduced graft survival. Consequently, high serum ferritin is regarded as a risk indicator for poor prognosis following liver transplantation^26^.

Alongside ferritin, other inflammatory biomarkers such as C-reactive protein (CRP) and interleukins—particularly IL-6—are elevated in liver disease and post-transplantation states. These markers may serve as confounders or complementary indicators when interpreting ferritin levels.

However, it is important to note that serum ferritin is a nonspecific marker. Its elevation does not always indicate hepatic iron overload, as it can also increase in response to inflammation, infection, malignancy, liver injury, and other systemic stressors^17^, ^27^. In liver transplant candidates and donors, elevated ferritin levels may reflect acute phase responses rather than true iron accumulation. Several studies have shown that serum ferritin correlates poorly with hepatic iron concentration (HIC), particularly in the presence of elevated CRP or liver inflammation^20^, ^27^.

Due to this limited specificity, serum ferritin is not commonly recommended as a standalone screening tool for hereditary hemochromatosis, especially in the absence of elevated transferrin saturation or genetic testing^20^.

Therefore, the use of serum ferritin as a surrogate marker of iron overload requires cautious interpretation, ideally combined with other markers (e.g., TSAT, hepcidin) or direct assessments (e.g., MRI-based iron quantification or liver biopsy in selected cases).

#### Transferrin

Transferrin (Trf), primarily synthesized in the liver, is the main iron transport protein in plasma, facilitating iron delivery to tissues^28^. It plays a protective role in maintaining iron homeostasis and preventing hepatic iron toxicity^28^. Studies indicate that moderate transferrin levels in donors correlate with favorable transplant outcomes, as transferrin effectively mobilizes iron, minimizing free iron accumulation and subsequently reducing oxidative stress and cellular damage. Adequate transferrin levels also appear to stabilize the postoperative immune environment, lowering the incidence of rejection and enhancing long-term graft survival^18^. Conversely, low transferrin levels may compromise iron transport, leading to free iron buildup in the liver, triggering oxidative stress via the Fenton reaction, which exacerbates cellular damage, delays recovery, and increases the risk of complications like acute rejection and chronic graft impairment.

One study analyzed clinical samples of liver transplantation provided by the Department of Liver Transplantation of Renji Hospital and found that the ubiquitination ligase E3 Huwe1/Mule was significantly down-regulated at the protein and mRNA levels after liver transplantation, suggesting the important effect of Huwe1/Mule on liver transplantation^29^. Huwe1/Mule alleviates acute liver injury by regulating the ubiquitination and degradation of transferrin receptor 1(TfR1) and inhibiting ferroptosis^30^. In another study, Huwe1 liver-specific knockout (Huwe1/Mule) mice were generated and subjected to hepatic ischemia-reperfusion (I/R), and a series of indicators of ferroptosis were measured. The results showed that HuWE1 / MULE liver-specific knockout aggravated the occurrence of ferroptosis and iron deposition in acute liver injure.

A retrospective analysis of 102 consecutive patients with acute liver failure found that patients with nonspontaneous recovery had higher ferritin levels and lower transferrin levels, which were associated with worse outcomes^17^.

#### Free Iron

The immune response after liver transplantation affects the success or failure of transplantation, and the free iron of donor liver is closely related to it. On the one hand, free iron in donor liver directly affects the function of immune cells, such as interfering with the signal transduction of T lymphocytes, inhibiting their immune response ability, affecting the phagocytosis and antigen presentation ability of macrophages, changing their cytokine secretion pattern, and increasing the risk of infection and rejection^9^, ^31^. On the other hand, free iron from donor liver indirectly affects the immune response by regulating the expression of iron metabolism-related proteins in immune cells, such as hepcidin expression, changing the iron concentration in the microenvironment of immune cells, and affecting the function and differentiation of immune cells, which is not good for long-term prognosis^32^.

Liver fibrosis affects the prognosis of liver transplantation recipients, and donor liver free iron plays a promoting role. The synthesis and degradation of ECM in normal liver are in balance, which can be broken by free iron in donor liver^33^. It directly stimulates the activation of hepatic stellate cells through oxidative stress, causing them to secrete a large number of ECM components^34^. It also activates MAPK and TGF-β signaling pathways, up-regulates the expression of ECM synthesis genes, inhibits the activity of degrading enzymes, aggravates ECM imbalance, accelerates liver fibrosis, and affects the long-term function of transplanted liver and the quality of life of recipients^35^. Increased mortality and morbidity.

#### Hepcidin

Hepcidin is a peptide hormone that regulates iron metabolism^36^. Synthesized predominantly in the liver, it exerts its regulatory function by binding to ferroportin, the sole known cellular iron exporter^37^. This binding event leads to the internalization and subsequent degradation of ferroportin, thereby controlling the release of iron from macrophages and enterocytes into the circulation. Hepcidin production is influenced by various factors, including iron levels, inflammation, and hypoxia, making it a crucial mediator in maintaining iron homeostasis within the body^38^.

Low hepcidin levels in the donor liver can precipitate inappropriate iron release, leading to iron overload in the transplanted liver^39^. A study involving 40 liver transplant patients demonstrated that those who received livers with subnormal hepcidin levels exhibited a more rapid iron accumulation in the graft during the initial post-transplant months. This iron overload was accompanied by increased oxidative stress, as evidenced by elevated levels of lipid peroxidation products in liver biopsies. Over time, the oxidative damage induced by iron overload contributed to the development of fibrosis. Significantly more patients in the low hepcidin group showed early-stage fibrosis compared to those with normal hepcidin levels. In some cases, the rapid progression of fibrosis led to impaired liver function and an elevated risk of graft failure within the first year post-transplant.

Conversely, extremely high hepcidin levels can result in iron sequestration and functional iron deficiency. This anemia was resistant to conventional iron supplementation, as the excessive hepcidin continued to sequester iron, impeding its utilization for hemoglobin synthesis. The chronic anemia had far-reaching consequences, affecting other organ systems. Reduced oxygen-carrying capacity led to compromised heart and kidney function, with some patients experiencing cardiac arrhythmias and renal insufficiency^40^.

Hepcidin also plays a role in modulating the immune response during liver transplantation. Abnormal hepcidin levels can influence the activation and function of immune cells, thereby affecting the risk of rejection^41^. In a study of 35 liver transplant patients, it was observed that those with higher hepcidin levels in the donor liver had a lower incidence of acute rejection episodes in the first six months post-transplant. The proposed mechanism involves hepcidin's regulation of iron availability, which is essential for immune cell proliferation and activation. By restricting iron, hepcidin may suppress the activation of T lymphocytes and macrophages, reducing the immune response against the transplanted liver. However, the long-term implications of this altered immune response and its relationship to chronic rejection and graft survival remain under investigation. Understanding these aspects is crucial for developing strategies to optimize the immune environment and improve long-term graft survival^42^.

In transplantation, moderate hepcidin levels in donors have been associated with favorable outcomes. These levels help regulate iron distribution, limiting excessive free iron and ROS formation^43^. By doing so, they protect the graft from oxidative damage and stabilize the immune environment post-transplant. This highlights the importance of maintaining hepcidin within an optimal range to enhance the success of liver transplantation.

#### Total Iron-Binding Capacity (TIBC)

Total iron-binding capacity reflects the plasma's potential iron-binding capacity and is used to assess overall iron metabolis^27^. Its role in transplantation remains ambiguous; elevated TIBC typically indicates low iron stores, which may reduce oxidative stress risk, yet may also signal insufficient iron availability, hindering hepatic function and recovery^44^. Thus, the prognostic value of TIBC requires further exploration.

#### Serum Iron

Serum iron represents circulating iron levels but has uncertain prognostic implications in transplantation. Moderate serum iron supports hepatocyte metabolism, promoting graft recovery, whereas excessive levels may elevate free iron and oxidative stress, increasing transplant failure risk^45^. Conversely, low serum iron may suggest inadequate iron stores, impairing liver regeneration. It is important to note that elevated serum iron does not necessarily indicate increased hepatic iron stores. In many liver diseases-such as viral hepatitis, alcoholic liver disease, and MAFLD--serum iron levels can rise due to hepatocyte damage, impaired iron storage, or hemolysis, rather than true iron overload in liver tissue^20^, ^27^.

In these contexts, serum iron reflects altered hepatic metabolism and release of stored iron into the circulation, often as a secondary effect of liver dysfunction. Therefore, serum iron should be interpreted with caution and ideally in conjunction with more specific indicators (e.g., hepatic iron concentration by imaging or biopsy, or ferritin and transferrin saturation) (see Table 2).

### Clinical measurement and availability of iron metabolism biomarkers

The routine assessment of iron metabolism in liver transplant patients primarily includes the measurement of serum ferritin, serum iron, transferrin, TIBC and transferrin saturation (TSAT). These are generally available in most clinical laboratories using automated immunoassays, colorimetric methods, or nephelometry, and results are typically reported within hours^27^, ^44^.

- Ferritin is measured via chemiluminescent or immunoturbidimetric assays.
- Serum iron is commonly measured via automated colorimetric assays.
- Transferrin can be measured directly via immunoassays or indirectly estimated through TIBC.
- TIBC is not measured directly but is commonly estimated by summing the serum iron and unsaturated iron-binding capacity (UIBC), which reflects the reserve iron-binding capacity of transferrin. The standard calculation is^27^:

TIBC (μg/dL) = Serum Iron (μg/dL) + UIBC (μg/dL)

- TSAT is then derived from these values as:

TSAT (%) = (Serum Iron / TIBC) × 100

These markers provide a practical estimation of transferrin-mediated iron transport and iron availability. Although TIBC is an indirect measure, it remains a valuable surrogate indicator of iron status, particularly when interpreted in conjunction with ferritin, transferrin, and inflammatory markers such as CRP.

In contrast, hepcidin measurement is not yet routinely available in most hospitals. It requires mass spectrometry or ELISA-based assays, and suffers from lack of assay standardization and reference ranges^36^, ^38^. Likewise, free iron or non-transferrin bound iron (NTBI) is technically challenging to measure and primarily used in research. These require high-sensitivity methods (e.g., HPLC, ultrafiltration-based assays), and are rarely accessible in routine clinical practice^22^, ^31^.

The limited clinical availability of hepcidin and free iron hampers the implementation of personalized iron regulation strategies in transplant settings. Without routine measurement, clinicians rely on surrogate markers (e.g., ferritin, TSAT), which can be confounded by inflammation. Thus, expanded access and standardization of these tests would greatly improve donor-recipient risk stratification and iron management protocols.

### Effect of recipient iron metabolism on the prognosis of transplantation

In recipients, high serum ferritin levels are associated with poorer post-transplant outcomes^46^. Elevated ferritin can exacerbate oxidative stress and immune activation, contributing to increased complications and reduced graft survival. Similarly, excessive free iron in recipients enhances ROS production, damaging cellular structures and delaying functional recovery. Elevated free iron also triggers inflammation and fibrosis, raising the risk of chronic graft dysfunction. High transferrin saturation (TSAT), indicating an abundance of bioavailable iron, is similarly linked to unfavorable outcomes, as it promotes oxidative stress and inflammatory responses, increasing the likelihood of liver injury and chronic rejection^18^.

Moderate hepcidin levels in recipients are protective, as they help regulate iron distribution, preventing excess free iron accumulation and associated oxidative damage^47^. Additionally, recipients with moderate transferrin levels tend to experience fewer inflammatory and rejection episodes, facilitating smoother graft integration and recovery. Conversely, the clinical relevance of TIBC remains uncertain, as high TIBC may indicate low iron stores, reducing overload risk yet potentially impairing cellular functions essential for recovery. Serum iron levels also lack definitive prognostic clarity; appropriate levels are essential for cellular metabolism, while imbalances, whether too high or too low, may disrupt graft health. Elevated soluble transferrin receptor (sTfR) levels, indicative of increased tissue iron demand, often reflect iron deficiency but may also signal compensatory responses to graft injury, necessitating further evaluation for accurate prognostication^48^, ^49^.

It is important to differentiate among various types of chronic liver disease (CLD) leading to transplantation, as iron metabolism may be altered differently depending on the underlying etiology. For instance, hereditary hemochromatosis causes primary iron overload, whereas chronic hepatitis C and MAFLD often feature secondary iron deposition due to hepatic inflammation and insulin resistance^6^, ^11^, ^46^. In contrast, alcoholic liver disease (ALD) is frequently associated with elevated ferritin and low transferrin due to both iron overload and systemic inflammation^10^. These disease-specific differences may influence the predictive value of iron biomarkers and the risk of graft dysfunction, infections, or post-transplant metabolic complications. Therefore, iron-related risk stratification should take into account the recipient's underlying liver disease type.

### Donor-recipient mismatched iron metabolisms on LT prognosis

A mismatch in iron metabolic levels between the donor and recipient may have a negative impact on transplant outcomes. Donor iron overload may lead to iron overload after transplantation in the recipient, increasing the risk of chronic graft dysfunction (CGD)^50^. In addition, iron overload may also increase the risk of damage and rejection reactions in the transplanted liver by promoting oxidative stress and inflammatory responses.

Therefore, the matching of iron metabolic levels between donors and recipients is extremely important for the recovery after liver transplantation. A higher degree of matching can significantly reduce the incidence of postoperative complications and improve the efficiency of liver function recovery; mismatch may increase the uncertainty of postoperative complications and long-term prognosis.

### Impact of infection and inflammation on iron biomarkers in donors and recipients

In both donors and recipients, systemic infection and inflammation during the perioperative period can markedly alter iron-related biomarkers. Specifically:

- Serum ferritin levels tend to increase as it is a positive acute-phase reactant, upregulated by inflammatory cytokines such as IL-6 and TNF-α^51^, ^52^.
- Transferrin levels decrease due to downregulation of hepatic synthesis during inflammation, contributing to a reduction in TIBC^27^.
- The decline in transferrin and TIBC may cause a falsely elevated transferrin saturation (TSAT), even when total body iron is not increased.

In donors, particularly those with terminal illness or ICU admission, undetected infections or systemic inflammatory responses can result in elevated ferritin and low transferrin, which may mimic iron overload and lead to misinterpretation of graft iron status. In recipients, post-transplant infections or sepsis can similarly elevate ferritin and distort TSAT, leading to inappropriate decisions regarding iron supplementation or chelation.

Therefore, we recommend that iron biomarkers in both donors and recipients be interpreted alongside inflammatory markers, such as CRP, IL-6, and clinical signs of infection, to avoid misdiagnosis of iron overload or deficiency under inflammatory stress^15^, ^17^, ^51^, ^52^.

### Hepatic iron assessment via liver biopsy

While circulating biomarkers such as ferritin, transferrin, and serum iron are commonly used to evaluate iron metabolism, they provide only an indirect assessment of hepatic iron load. In contrast, quantitative hepatic iron analysis via liver biopsy—using either Perls' Prussian blue staining for histological grading or biochemical quantification (e.g., μmol/g dry weight)—offers a direct measurement of hepatic iron content.

Liver biopsy remains the gold standard for diagnosing hepatic iron overload, particularly in conditions such as hereditary hemochromatosis or secondary hemosiderosis^53^. In the transplant setting, pre-procurement donor liver biopsy can help identify excessive hepatic iron accumulation, which may contribute to oxidative stress, promote ferroptosis, and impair graft function post-transplant^54^. Histological scoring systems such as the Scheuer scale or Deugnier scale can semi-quantitatively assess hepatic iron deposition, providing an objective measure complementary to serum ferritin or TSAT.

However, the routine use of liver biopsy in donor evaluation remains controversial. It is invasive, time-consuming, and often impractical in the setting of deceased donation. Moreover, interobserver variability in grading and sampling error can affect reliability. In recipients, liver biopsy may be useful post-transplant to investigate unexplained graft dysfunction, particularly when iron overload is suspected but serum markers are confounded by inflammation or hemolysis^55^.

### Ferroptosis: an iron-dependent mechanism in liver injury

Ferroptosis is a recently defined form of regulated cell death, driven by iron-catalyzed lipid peroxidation and distinct from apoptosis or necrosis^7^. In the context of liver transplantation, accumulating evidence suggests that ferroptosis plays a critical role in hepatic ischemia-reperfusion injury (IRI), a key determinant of graft dysfunction.

During ischemia, disruption of cellular antioxidant defenses (e.g., glutathione depletion and GPX4 inactivation), combined with increased labile iron and ROS, triggers peroxidation of polyunsaturated phospholipids. This initiates ferroptotic cell death, contributing to hepatocellular damage and inflammatory amplification.

Animal models have shown that targeting ferroptosis with inhibitors such as ferrostatin-1 or liproxstatin-1 can attenuate hepatic IRI and improve graft outcomes. Thus, ferroptosis represents a mechanistically distinct and potentially modifiable contributor to transplant-related liver injury^56^.

In sum, although liver biopsy is not routinely employed in all transplant cases, its role remains important in selective scenarios—particularly when serum-based markers are inconclusive or when accurate quantification of hepatic iron is required to guide clinical decision-making.

## Potential Mechanism for Iron Metabolic Disorder and Hepatic Ischemia-Reperfusion Injury

**Figure 1:**
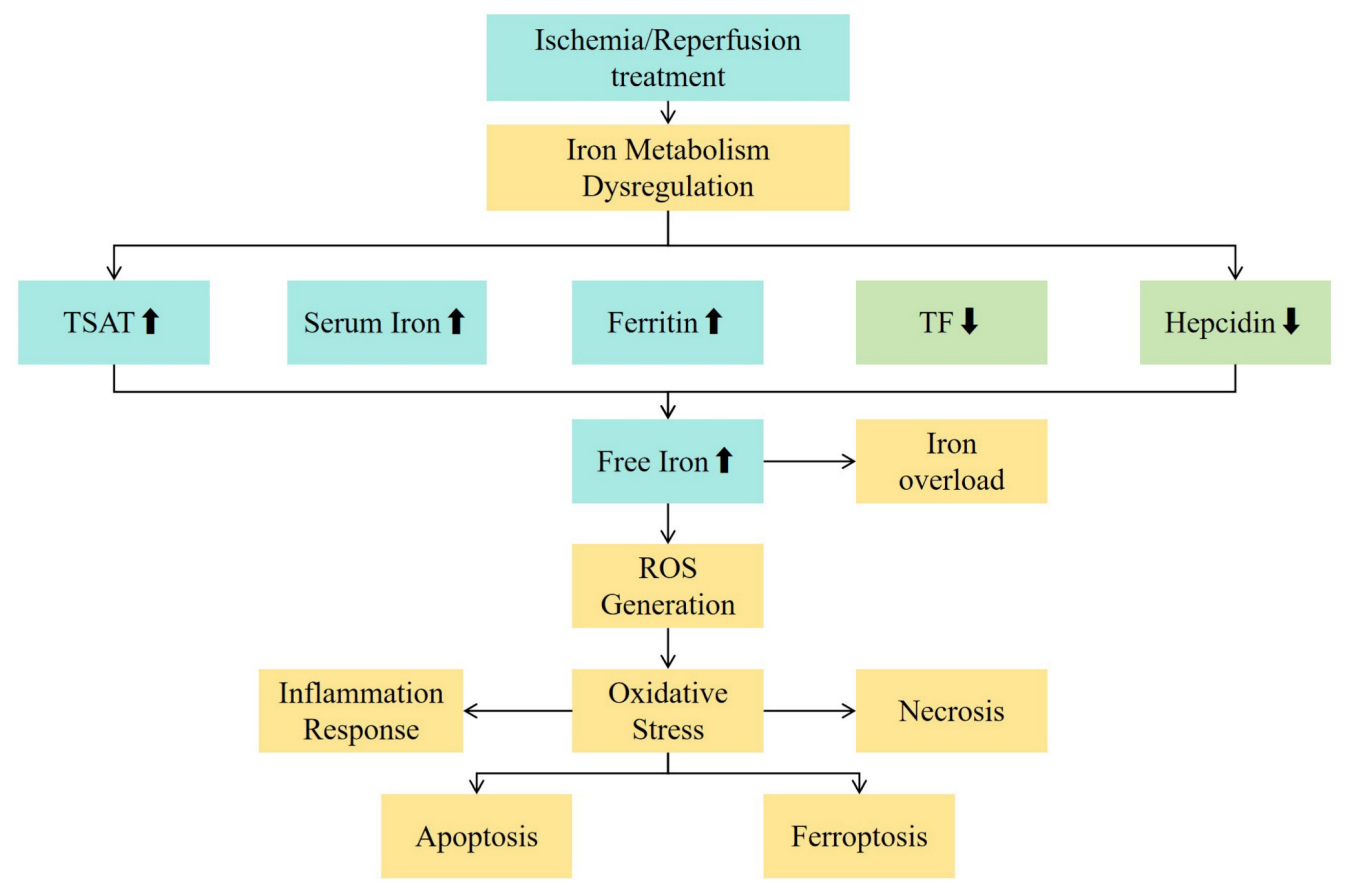
The relationship between iron metabolism indicators and the mechanism of ischemia-reperfusion injury. Abbreviations: TSAT - transferrin saturation; TF - transferrin; ROS - reactive oxygen species.

IRI is the most fundamental mechanism of damage in liver transplantation^57^, ^58^. IRI occurs when the blood supply to the liver is interrupted (ischemia) and blood flow is restored (reperfusion), triggering an intense oxidative stress response, inflammatory reaction, and cellular damage^59^. It is an important cause of transplant liver dysfunction and rejection reactions. During the IRI process, the large amount of ROS and iron metabolism abnormalities may exacerbate adverse reactions such as lipid peroxidation of cell membranes, mitochondrial damage, and cell death, affecting the functional recovery of the liver after transplantation^60^. In addition to classical oxidative mechanisms, iron overload may promote ferroptosis, a non-apoptotic form of regulated cell death. The accumulation of lipid peroxides and failure of antioxidant defenses, particularly GPX4 inhibition, are key triggers. Ferroptosis may thus be a central link between iron metabolism disturbance and hepatocyte injury during IRI.A multi-omics analysis has revealed a crosstalk between ferroptosis and peroxisomes on steatotic graft failure after liver transplantation, providing insights into the mechanisms underlying graft dysfunction^61^. See Figure 1 for the mechanism diagram.

### Serum Ferritin

As the main intracellular iron storage form, serum ferritin has its structure damaged when cells are damaged, resulting in the dissociation of the ferritin-iron complex and the release of iron^62^. This abnormal release of intracellular iron is a key initiating link in iron metabolism disorders in IRI.From the cellular level, during the ischemia process, the function of mitochondria is impaired, with changes in their membrane potential and increased permeability, which may prompt the release of iron from ferritin in mitochondria. Meanwhile, endoplasmic reticulum stress may also be involved. When the function of the endoplasmic reticulum is disrupted, the ferritin stored therein will also release iron into the cytoplasm, thus affecting the intracellular iron balance^25^.

Clinical studies have found that among patients who experienced IRI after liver transplantation, those with significantly elevated serum ferritin levels showed more severe damage in liver tissue pathology^63^. For example, a study involving 100 liver transplantation patients indicated that patients with serum ferritin levels more than twice the upper limit of the normal range had an average proportion of liver necrosis area reaching about 30% within 72 hours after surgery, while in patients with normal serum ferritin levels, the proportion of liver necrosis area was only about 10%^64^.

Experimental studies have also confirmed elevated serum ferritin levels in animal models with IRL. In a rat model of hepatic IRI, after exogenous administration of ferritin to increase the serum ferritin level, a significant increase in the production of ROS in liver tissues was observed, along with a significant decrease in the activities of antioxidant enzymes such as superoxide dismutase (SOD), and an approximately 60% increase in the content of malondialdehyde (MDA), a lipid peroxidation product^65^. This shows that iron overload caused by the increase in serum ferritin can trigger a strong oxidative stress response, severely damage the liver's antioxidant defense system, and aggravate IRI injury^66^.

A low serum ferritin level may imply insufficient iron reserves in the body. In the context of hepatic IRI, insufficient iron reserves may limit the utilization of iron by hepatocytes during the recovery stage^25^. For example, in some liver transplantation recipients with malnutrition or long-term iron deficiency, lower serum ferritin levels were associated with slow recovery of liver function after surgery. Studies have found that the activity of cytochrome P450 enzymes in hepatocytes of these patients was reduced, affecting drug metabolism and detoxification functions and leading to an increased incidence of adverse drug reactions after surgery^67^.

From the perspective of cellular metabolism, a decrease in ferritin levels may weaken the intracellular iron buffering capacity. When cells need iron to participate in energy metabolism (such as iron-sulfur cluster proteins in the tricarboxylic acid cycle) or the synthesis of iron-containing enzymes, insufficient iron supply will lead to cellular metabolic disorders^68^. In a mouse model of hepatic IRI, mice with ferritin gene knockout exhibited obvious mitochondrial dysfunction, reduced ATP production, and increased apoptosis, suggesting that a decrease in serum ferritin levels has an adverse impact on the normal metabolism and survival of liver cells^69^.

### Hepcidin

Hepatic IRI can trigger a series of inflammatory responses. The release of inflammatory factors such as interleukin-6 (IL-6) and tumor necrosis factor-α (TNF-α) will interfere with the normal synthesis and secretion of hepcidin^51^. Under normal circumstances, hepcidin is mainly synthesized in the liver and binds to ferroportin on the cell membrane, promoting its internalization and degradation, thereby reducing the release of intracellular iron^52^. During IRI, the activation of inflammatory signaling pathways may inhibit the synthesis of hepcidin, weakening the inhibitory effect on ferroportin. At the cellular level, hypoxia-inducible factor-1α (HIF-1α) is activated in an ischemic environment and can regulate the expression of hepcidin^70^. HIF-1α may interact with the binding sites in the promoter region of the hepcidin gene to inhibit the transcription of hepcidin, resulting in changes in hepcidin levels and thus affecting the output of intracellular iron^56^.

In some special cases, such as the repair stage after hepatic IRI, a moderate increase in hepcidin may have a protective effect. Studies have found that in a mouse model of hepatic IRI, through gene therapy to moderately increase hepcidin after injury, iron deposition in liver tissues could be reduced^71^. Specifically, the increase in hepcidin reduced the activity of ferroportin, preventing excessive iron from flowing out of cells, thereby reducing iron-mediated oxidative damage. The iron content in liver tissues decreased by about 30% 72 hours after injury, and the degree of inflammatory cell infiltration and hepatocyte necrosis also decreased.

However, if hepcidin is continuously and excessively increased, it will lead to excessive iron retention in cells. In clinical studies, it has been found that some patients with chronic liver diseases who experienced IRI after liver transplantation had chronically high hepcidin levels. These patients showed symptoms of functional iron deficiency, such as reduced hemoglobin synthesis, inhibited erythropoiesis, and the appearance of anemia symptoms^72^. Meanwhile, due to excessive iron retention in cells, the activities of iron-containing enzymes in the mitochondrial respiratory chain were affected, resulting in cellular energy metabolism disorders and poor liver function recovery^73^.

A decrease in hepcidin level is a common phenomenon in hepatic IRI. In the early ischemic stage, a rapid decrease in hepcidin will lead to a relative increase in the activity of ferroportin and a large amount of iron release from cells. In a study on a rat model of hepatic IRI, the hepcidin level decreased significantly 30 minutes after ischemia, and then the release of intracellular iron increased^74^. The concentrations of free iron in the blood and liver tissues increased by about 50% and 40%, respectively, within 1 hour after reperfusion. These free irons generate a large amount of ROS through the Fenton reaction, triggering oxidative stress and leading to hepatocyte damage and death^75^.

From an immunological perspective, a low hepcidin level will change the iron uptake and utilization of immune cells^76^. In liver transplantation patients, those with lower hepcidin levels had a higher incidence of acute rejection in the early postoperative period. This is because immune cells (such as T lymphocytes and macrophages) can obtain iron more easily in a low hepcidin environment, thereby enhancing their activation and proliferation abilities and strengthening the immune attack on the transplanted liver^77^.

### Transferrin

During hepatic IRI, damage to vascular endothelial cells is one of the early events. Damaged vascular endothelium will lead to increased vascular permeability, affecting the normal transport of transferrin in blood vessels^78^. Meanwhile, hepatocyte damage will change the expression and function of transferrin receptors on the cell membrane. Under normal circumstances, after transferrin binds to its receptors, iron is transported into cells through endocytosis. During IRI, a reduction in receptors or functional impairment will hinder the uptake of the transferrin-iron complex^79^.

At the molecular level, oxidative stress induced by ischemia-reperfusion can cause structural changes in transferrin. ROS can oxidize the amino acid residues of transferrin, affecting its binding ability to iron and receptors. In addition, the disorder of intracellular signaling pathways, such as the activation of the protein kinase C (PKC) pathway, may also interfere with the normal transport and uptake mechanism of transferrin^80^.

An increase in transferrin level may be a compensatory response of the body to hepatic IRI. However, excessively elevated transferrin may aggravate liver damage. In a prospective study on liver transplantation patients, it was found that in some patients, the transferrin level continued to increase after surgery. These patients had significantly higher iron deposition in liver tissues within one week after surgery than those with normal transferrin levels. Liver histopathological examination showed that the iron deposition areas were accompanied by obvious inflammatory cell infiltration and hepatocyte swelling. Further studies found that the elevated transferrin level was associated with increased levels of inflammatory factors (such as IL-6 and C-reactive protein) in serum, suggesting that the increase in transferrin may aggravate hepatic IRI by promoting iron deposition and inflammatory responses^81^.

From the perspective of immune regulation, a high transferrin level may affect the function of immune cells. In *in vitro* experiments, when transferrin was added to a culture system containing immune cells (T lymphocytes and monocytes), the expression of activation markers on immune cells increased, and the secretion of cytokines also increased^82^. In patients with hepatic IRI after liver transplantation, a high transferrin level may change the local immune environment in the liver through a similar mechanism and increase the risk of rejection.

A decrease in transferrin level will lead to insufficient iron transport. During the recovery stage after hepatic IRI, hepatocytes need sufficient iron for repair and regeneration. For example, in a mouse model of hepatic IRI, mice with transferrin gene deficiency showed obvious delays in liver function recovery after injury. The iron content in the hepatocytes of these mice was lower, resulting in decreased activities of cytochrome P450 enzyme systems and iron-containing enzymes in the mitochondrial respiratory chain, as well as impaired energy metabolism and detoxification functions^67^. Meanwhile, due to insufficient iron transport, erythropoiesis was also affected, and anemia symptoms appeared, further affecting the systemic oxygen delivery and liver recovery^83^.

From the perspective of infection risk, the iron-binding ability of transferrin can limit the access of pathogens to iron. In clinical liver transplantation patients, those with lower transferrin levels had a higher incidence of postoperative infections. This is because when transferrin is insufficient, the free iron in the blood increases, providing more iron sources for bacteria, fungi and other pathogens and facilitating their growth and reproduction.

### Serum iron

During hepatic IRI, the intracellular iron metabolism balance is disrupted. Ischemia leads to cell damage and impairs the integrity of the cell membrane, causing the release of intracellular iron into the extracellular space and then into the bloodstream, resulting in an increase in serum iron levels^84^. Meanwhile, changes in the functions of transferrin and its receptors will also affect the serum iron level. If transferrin cannot transport the released iron into cells in a timely manner, serum iron will accumulate in the blood.

At the cellular metabolism level, organelles such as mitochondria and the endoplasmic reticulum have their functions disrupted during the ischemia-reperfusion process, and the iron stored therein will also be released^85^. The iron in mitochondria participates in the electron transfer of the respiratory chain. When mitochondria are damaged, iron is released into the cytoplasm and then enters the bloodstream. The endoplasmic reticulum is also involved in the utilization of iron in the process of protein synthesis and folding, and its functional abnormalities will lead to the release of iron and changes in serum iron levels.

An increase in serum iron level is one of the important causes of oxidative stress in hepatic IRI. Clinical studies have found that in liver transplantation patients, those with postoperative serum iron levels higher than the upper limit of the normal range had significantly elevated oxidative damage indicators (such as MDA and 8-hydroxydeoxyguanosine) in liver tissues^86^. For example, in a group of 50 liver transplantation patients who experienced IRI, the MDA content in the liver tissues of patients with serum iron levels higher than 200 μg/dL was about twice that of patients with normal serum iron levels^87^. These oxidative damage products can cause lipid peroxidation, protein oxidation, and DNA damage in hepatocytes, thereby aggravating liver damage.

From the perspective of infection, high serum iron provides a rich iron source for pathogens. After liver transplantation, the patient's immune system is in a relatively fragile state, and high serum iron will increase the risk of bacterial and fungal infections. A study on post-liver-transplantation infections found that patients with serum iron levels higher than 150 μg/dL had an approximately 40% higher incidence of infections within one month after surgery than those with normal serum iron levels. This is because the growth and reproduction of many pathogens depend on iron, and a high serum iron environment is conducive to their survival and spread in the body^88^.

A decrease in serum iron level will lead to iron deficiency, affecting the normal functions of hepatocytes. Many enzymes in hepatocytes (such as cytochrome P450 enzymes and iron-containing antioxidant enzymes) require iron as a cofactor^89^. In liver transplantation patients, those with lower serum iron levels had slow recovery of liver function. For example, in patients with serum iron levels lower than 80 μg/dL, the time for postoperative serum transaminases (ALT and AST) to return to normal was extended by about one week compared to patients with normal serum iron levels^90^. This is because iron deficiency leads to a decrease in the activities of these enzymes in hepatocytes, affecting the liver's metabolism and detoxification functions.

From the perspective of immune function, low serum iron will affect the activity of immune cells. Iron is an essential element for the metabolism and proliferation of immune cells (such as T lymphocytes and macrophages). In animal experiments, after reducing the serum iron level in mice through dietary control and then performing hepatic IRI surgery, it was found that the proliferation ability of T lymphocytes in mice decreased, the phagocytic function of macrophages weakened, and the body's resistance to pathogens decreased, increasing the risks of infection and inflammatory responses.

### Total Iron-binding Capacity (TIBC)

TIBC mainly depends on the levels of transferrin and other iron-binding proteins. During hepatic IRI, inflammatory responses and metabolic changes in the body can affect the synthesis and functions of these proteins. Inflammatory factors such as IL-6 and tumor necrosis factor-α (TNF-α) can regulate the synthesis of transferrin. When inflammation is triggered by IRI, it may lead to an increase in transferrin synthesis, thereby increasing TIBC. Meanwhile, liver cell damage can affect the secretion and metabolism of iron metabolism-related proteins, changing the total amount of iron-binding proteins in the blood and thus influencing TIBC.

At the cellular level, factors such as hypoxia and oxidative stress can alter the structures and functions of intracellular iron-binding proteins. For example, endoplasmic reticulum stress may cause misfolding of some intracellular iron-binding proteins, resulting in the loss of their normal iron-binding abilities. These changes will be fed back to the blood and affect the level of TIBC.

An increase in TIBC may indicate iron deficiency in the body or an increased demand for iron-binding capacity. In the context of hepatic IRI, a high TIBC may be a manifestation of the body's attempt to compensate for iron deficiency. For instance, in some liver transplantation patients with iron deficiency caused by chronic blood loss or malnutrition, TIBC is elevated. However, if this iron-deficient state persists, it will affect liver recovery. In these patients, due to iron deficiency, the activities of iron-containing enzymes in hepatocytes are reduced, and energy metabolism and anabolism are affected, resulting in slow recovery of liver function. Meanwhile, a high TIBC may change the distribution of iron between immune cells and pathogens, making it more difficult for immune cells to obtain iron and thus affecting immune function. In clinical studies, it has been found that patients with elevated TIBC have a relatively higher infection rate after liver transplantation, which may be related to the suppression of immune cell functions.

A decrease in TIBC may be related to iron overload or abnormal iron metabolism. When TIBC is reduced, the binding ability of free iron in the blood is weakened, which may lead to the accumulation of free iron in the body. In an animal model of hepatic IRI, after inhibiting the synthesis of transferrin by chemical drugs to reduce TIBC, it was observed that iron deposition in liver tissues increased significantly and oxidative stress damage was aggravated. The content of MDA in liver tissues increased by about 50%, and the apoptotic index of hepatocytes increased by 30%. This indicates that the accumulation of free iron caused by the decrease in TIBC aggravates hepatic IRI.

From an immunological perspective, low TIBC may make it easier for pathogens to obtain iron, thus promoting their growth and causing infections. In *in vitro* experiments, when bacteria were cultured together with serum with low TIBC, it was found that the growth rate of bacteria was significantly faster than that in serum with normal TIBC. In liver transplantation patients, low TIBC may increase the risk of postoperative infections, especially when the liver has already suffered from IRI and the immune function is relatively weak.

### Free iron

During ischemia, the energy metabolism of the donor liver cells is disturbed, the cell membrane is damaged, and free iron is released from ferritin. During reperfusion, free iron produces hydroxyl radicals through the Fenton reaction, which causes lipid peroxidation and damages cell membranes, proteins and nucleic acids. It also activates the NF-κB signaling pathway, promotes the release of inflammatory factors, attracts immune cell infiltration, and forms a vicious circle to aggravate liver IRI and affect the early prognosis.

In both hereditary hemochromatosis and liver transplantation-related ischemia-reperfusion injury (IRI), the Fenton and Haber-Weiss reactions are central to amplifying oxidative stress via iron-catalyzed hydroxyl radical generation^91^. In hemochromatosis, chronic systemic iron overload leads to progressive hepatic iron deposition, driving persistent lipid peroxidation, mitochondrial dysfunction, and ultimately fibrosis or hepatocellular carcinoma^92^.

In contrast, during liver transplantation, especially with marginal grafts such as DCD or steatotic livers, acute ischemia and subsequent reperfusion rapidly mobilize labile iron from injured hepatocytes and Kupffer cells. This promotes a sudden surge in ROS through the same chemical reactions, leading to acute hepatocellular injury^93^.

Despite differences in chronicity and etiology, both processes establish a self-reinforcing oxidative vicious cycle: excess iron promotes ROS generation via the Fenton/Haber-Weiss pathway, which in turn causes more cellular damage, further disrupts iron regulation (e.g., hepcidin suppression), and amplifies injury. This common biochemical cascade provides a mechanistic basis for targeting iron and ROS in both genetic and transplant-related liver injury (see Table 3).

## Management of Iron Metabolism in Clinical Practice for Liver Transplant Recipients

In liver transplantation, managing iron metabolism in recipients is crucial for optimizing transplant success and improving postoperative recovery. Both preoperative and postoperative iron regulation play significant roles in mitigating complications related to iron overload or deficiency, which can impact graft function, immune response, and overall patient outcomes^94^.

### Preoperative iron management

Prior to liver transplantation, a comprehensive assessment of iron metabolic indicators in recipients is a crucial first step. This involves measuring indicators such as serum ferritin, serum iron, TIBC, transferrin saturation, and hepcidin. Through these indicators, the iron storage status, transport capacity, and the normality of the regulatory mechanism within the recipient's body can be accurately understood. For example, an elevated serum ferritin level may suggest iron overload, while low serum iron and high TIBC may imply an iron-deficient state^95^. Such information plays a key guiding role in formulating personalized iron management strategies subsequently.

#### Management of iron overload

Dietary Adjustment: For recipients diagnosed with iron overload, the intake of iron-rich foods, such as red meat, liver, and beans, should be strictly restricted. It is recommended to increase the consumption of foods rich in vitamin C. Although vitamin C can promote iron absorption, in the case of iron overload, an appropriate amount of vitamin C helps maintain the body's antioxidant balance and reduces the risk of oxidative damage caused by iron overload. For instance, consuming more fruits like oranges, lemons, strawberries, and vegetables such as broccoli and green peppers.

Pharmacological Treatment: In certain circumstances, iron chelators may be required to reduce the body's iron level. Iron chelators can bind to the excess iron in the body to form complexes that can be excreted from the body, thereby reducing the damage of iron overload to the liver and other organs. Commonly used iron chelators, such as deferoxamine, etc., should have their dosage and treatment course adjusted under the close monitoring of doctors according to the specific situation of the recipient to ensure safe and effective reduction of iron overload and avoid adverse reactions such as iron deficiency caused by excessive chelation.

#### Intervention for iron deficiency

Dietary Supplementation: If the recipient has iron deficiency, the diet should be supplemented with iron-rich and easily absorbable foods, such as lean meat, fish, eggs, and green leafy vegetables. Meanwhile, it is advisable to combine them with foods rich in vitamin C to promote iron absorption. For example, pairing lean meat with oranges can improve the bioavailability of iron^96^.

Iron Supplementation: For recipients with severe iron deficiency, dietary adjustment alone may not meet the body's iron requirements. At this time, oral iron supplements need to be considered. When choosing iron supplements, preparations with less gastrointestinal irritation should be preferred, and they should be taken according to the appropriate dosage and frequency as prescribed by doctors. Generally, ferrous sulfate and ferrous fumarate are commonly used oral iron supplements. They can be taken together with vitamin C to enhance the absorption effect of iron. During the process of iron supplementation, serum ferritin, hemoglobin, and other indicators should be regularly monitored to evaluate the effect of iron supplementation and avoid iron overload^97^.

### Postoperative iron management

After liver transplantation, the recipient's iron metabolism changes during recovery. Firstly, close monitoring of iron metabolic indicators such as serum ferritin and hepcidin is necessary. The frequency, set according to the recipient's situation, helps detect disorders like those caused by surgery, immunosuppressive drugs, or liver function changes, enabling timely treatment adjustments^95^.

Infections pose a significant risk post-transplant and are intertwined with iron metabolism. Bacterial infections can increase hepcidin, altering iron availability. When infections occur, besides anti-infective treatment, iron metabolism should be monitored^99^. If hepcidin changes lead to iron deficiency affecting the immune system, appropriate adjustments like cautious iron supplementation may be considered to avoid worsening the infection.

The liver's recovery is vital for iron metabolism normalization. Nutritional support, providing necessary nutrients, benefits both liver function and iron metabolism. For example, branched-chain amino acids help liver protein synthesis and iron handling^98^. Also, iron supplementation or chelation strategies should be adapted to different liver function recovery stages to prevent iron imbalances and maintain long-term liver health.

### Role of liver biopsy in iron assessment

While non-invasive serum biomarkers such as ferritin, TSAT, and hepcidin are widely used for assessing systemic iron status, liver biopsy remains the gold standard for evaluating hepatic iron concentration, particularly in ambiguous or complex clinical scenarios^20^. Tissue iron can be assessed by semiquantitative histological staining (e.g.,Perls' Prussian blue stain) or by quantitative biochemical measurement of hepatic iron concentration (HIC), typically expressed as μmol/g dry liver weight^6^, ^20^.

In liver transplantation, liver biopsy may be valuable in cases of suspected hereditary hemochromatosis, unexplained graft dysfunction, or when serum ferritin is elevated but confounded by inflammation. However, its routine use is limited due to its invasive nature, bleeding risk, and potential for sampling error due to heterogeneous iron distribution^6^.

Therefore, while liver biopsy is not routinely recommended for all transplant candidates or recipients, it can serve a diagnostic adjunct in selected patients where serum iron markers are unreliable or where histological confirmation of iron overload is necessary.

In conclusion, the preoperative and postoperative iron management of liver transplant recipients is complex. Considering various factors and implementing precise monitoring and interventions are key to maintaining iron metabolism balance, optimizing transplantation results, and ensuring the recipient's long-term well-being.

## Future Research Directions

Large-scale prospective studies are essential for comprehensively understanding the role of iron metabolism in chronic liver disease and liver transplantation. By longitudinally following a substantial number of cases, a more precise evaluation of the impact of donor and recipient iron metabolism levels on prognosis, as well as the correlation between iron metabolic aberrations and post-transplant complications, can be achieved. Additionally, such studies are conducive to determining the optimal timing and strategies for managing iron metabolism, thereby enhancing the transplant protocol.

### Elucidating molecular mechanisms

A primary focus lies in deciphering the molecular pathways through which abnormal iron metabolism induces complications following liver transplantation. For instance, further characterizing the intracellular signaling alterations in the liver under iron overload or deficiency conditions and their intersections with inflammation, oxidative stress, and apoptosis, which lead to complications like IRI, bile duct damage, infection, and tumor recurrence. Leveraging advanced proteomic, metabolomic, and gene editing tools to elucidate these intricate molecular networks in cellular and animal models will uncover potential therapeutic targets, providing a robust theoretical foundation for targeted interventions.

### Enhancing monitoring methodologies

Exploring more accurate and individualized approaches for monitoring iron metabolism is of utmost significance. While current iron metabolism indicators offer some insights, they have limitations in reflecting the local hepatic iron metabolism and its dynamic changes. Future research could center on developing novel imaging modalities or biomarkers. For example, optimizing liver iron quantification techniques based on magnetic resonance imaging to more precisely assess iron distribution and content in the liver. Alternatively, identifying circulating biomarkers that specifically reflect the functional state of liver cell iron metabolism and integrating them with the existing index system to construct a more comprehensive and accurate monitoring model. This would enable real-time and dynamic monitoring of liver transplant recipients' iron metabolism, facilitating early detection and personalized treatment plan adjustments.

### Optimizing management strategies

Optimizing the iron management strategy for liver transplant recipients is a pressing clinical need. This involves further investigations into the optimal timing, dosage, and duration of iron supplementation or chelation therapy across different stages (preoperative, perioperative, and long-term postoperative). The impact of these interventions on long-term graft function and recipient quality of life also requires evaluation. Considering the complex interplay between immunosuppressive agents and iron metabolism, prospective clinical studies should be conducted to explore how to optimize the iron metabolism balance by adjusting the immunosuppressive regimen. This would mitigate the occurrence of iron metabolism disorders and related complications induced by immunosuppressive agents, presenting new avenues for improving liver transplantation outcomes.

## Conclusion

Iron metabolism plays a crucial role in determining the outcome of liver transplantation. The level of iron metabolism in both donors and recipients significantly affects the recovery after transplantation. Iron overload and iron deficiency may lead to complications such as graft dysfunction, oxidative stress and immune response. Effective management of iron homeostasis is essential to improve graft survival, reduce postoperative complications, and ensure better long-term outcomes for transplant patients. Clinically, it is essential to enhance the understanding of the management of iron metabolism before and after liver transplantation, and routine preoperative iron biomarker assessments, including serum ferritin, transferrin saturation, and hepcidin levels, should be performed to develop individualized treatment plans. After surgery, regular monitoring of iron status and timely iron chelation or replacement therapy can help mitigate the risk of iron overload or deficiency and promote a smoother recovery and better overall prognosis. Future translational work should focus on validating ferroptosis as a therapeutic target in liver transplantation. Strategies aimed at modulating the labile iron pool, enhancing GPX4 activity, or using ferroptosis inhibitors may provide novel means to mitigate ischemia-reperfusion injury and improve graft outcomes. By integrating these strategies into clinical practice, medical professionals can optimize iron metabolism management and ultimately improve the success rate and quality of life of liver transplant recipients. Further research into individualized iron metabolism management protocols is needed to refine these approaches and enhance transplant care.

## Figures and Tables

**Table 1:** Literature characteristics and original reports of clinical studies on liver transplantation.

Author, Publication year (Ref)	Country	Study Design	Markers of Iron Metabolism	Sample Size	Donor/Recipient	Results Summary
Walker,2009^21^	Australia	Retrospective analysis	Ferritin	322	Recipient	Serum ferritin concentration is an independent predictor of mortality-related and liver-related clinical events.
Weismuller,2011^18^	Germany	Retrospective analysis	Ferritin/transferrin saturation (TFS)	328	Donor	SF concentration 365 μg/L in combination with TFS <55% before LT is an independent risk factor for mortality following LT
Al-Freah,2013^24^	Britain	Retrospective analysis	Ferritin	1079	Donor/Recipient	A limited role for SF as a prognostic indicator for pre- or post-transplant survival.
Wakiya,2015^19^	Japan	Retrospective analysis	Ferritin	98	Donor	A high SF level in the donor is associated with the risk of further acute reactions, such as IRI, in the recipient
Chow,2017^15^	America	Prospective study	Ferritin/hepcidin/iron	128	Recipient	Time-dependent associations showed that increasing ferritin, ferritin slope, and hepcidin, as well as decreasing iron and iron slope were associated with the development of infection after LT.
Yamada,2020^22^	Japan	Retrospective analysis	Ferritin	202	Donor	A high serum ferritin level, a marker of iron overload, of the donor is an independent risk factor for liver damage after LT

LT: liver transplantation; SF: serum ferritin

**Table 2:** Relationship between iron metabolism indicators and transplantation prognosis in liver transplant donors.

Iron Metabolism Indicator	Functional Description	Level in Donor	Prognostic Impact	Associated Mechanism	Reference
Ferritin	Primary iron storage protein	High	Increases oxidative stress, delays graft recovery	Promotes ferroptosis and inflammation	15, 16, 22
Free Iron	Active iron ion, prone to oxidation	High	Increases rejection risk, causes graft injury	Enhances ROS generation, leads to cellular necrosis	32, 33
Transferrin	Iron transport protein in plasma	Moderate	Promotes recovery, reduces complications	Stabilizes immune environment	18, 58
Hepcidin	Regulates iron absorption and release	Moderate	Reduces oxidative damage, stabilizes graft function	Inhibits free iron production	39
Total Iron-Binding Capacity	Potential iron-binding capacity in plasma	Neutral	Uncertain, may indicate iron overload or deficiency	Requires further investigation	27, 44

**Table 3:** The role of iron metabolism in ischemia-reperfusion injury during liver transplantation.

Phase	Involved Factors	Mechanism	Outcome
Ischemic phase	Ferritin release, Fe²⁺ accumulation	Iron ions accumulate intracellularly during hypoxia	Sets the stage for ROS generation on reperfusion
Early reperfusion	Fenton reaction, iron overload	Fe²⁺ + H₂O₂ → •OH via Fenton chemistry	Oxidative stress, lipid peroxidation
Mid-reperfusion	Ferroptosis pathway activation	Inactivation of GPX4 + iron-dependent lipid peroxidation	Programmed hepatocyte death, graft injury
Systemic response	High TSAT, elevated CRP	Acute phase response alters iron biomarker interpretation	Affects clinical assessment and timing of intervention

TSAT: transferrin saturation; ROS: reactive oxygen species; CRP: C-reactive protein; GPX4: glutathione peroxidase

## Abbreviations

LT: liver transplantation
SF: serum ferritin
ROS: reactive oxygen species
CGD: chronic graft dysfunction
Trf: transferrin
TfR1: transferrin receptor 1
I/R: ischemia-reperfusion
TIBC: total iron-binding capacity
TSAT: transferrin saturation
sTfR: soluble transferrin receptor
IRI: ischemia-reperfusion injury
SOD: superoxide dismutase
MDA: malondialdehyde
IL-6: interleukin-6
TNF-α: tumor necrosis factor-α
HIF-1α: hypoxia-inducible factor-1α
PKC: protein kinase C
AST: aspartate aminotransferase
ALT: alanine transaminase
NF-κB: nuclear factor kappa-B
CNIs: Calcineurin Inhibitors
DNA: deoxyribonucleic acid
ECM: extracellular matrix
MAPK: mitogen-activated protein kinase
MAFLD: metabolic-associated fatty liver disease
ALD: alcoholic liver disease
CLD: chronic liver disease
NTBI: non-transferrin bound iron
HIC: hepatic iron concentration
GPX4: glutathione peroxidase 4
DCD: donation after circulatory death
CRP: C-reactive protein
UIBC: unsaturated iron-binding capacity
